# Treatment With Phosphodiesterase 5 Inhibitors and Long‐Term Outcomes in Patients Undergoing Coronary Angiography and Cardiac Catheterization

**DOI:** 10.1002/ccd.31624

**Published:** 2025-05-27

**Authors:** Sumon Roy, Annette Min, Edward O. McFalls, Robert A. Perera, Fadi N. Salloum, Ion S. Jovin

**Affiliations:** ^1^ McGuire Veterans Administration Medical Center Richmond Virginia USA; ^2^ Virginia Commonwealth University Richmond Virginia USA

**Keywords:** cardiac catheterization, coronary angiography, major adverse cardiovascular events, outcomes, phosphodiesterase 5 inhibitors

## Abstract

**Background:**

The effects of phosphodiesterase 5 (PDE5) inhibitors on the incidence of long‐term outcomes in patients with cardiovascular disease are not well understood. Objective: We studied the association between PDE5 inhibitor therapy and the incidence of adverse cardiovascular major adverse cardiovascular events (MACE) in patients undergoing coronary angiography and intervention.

**Methods:**

We studied 4582 consecutive patients undergoing coronary angiography and intervention. The incidence of MACE at 1 year, defined as urgent revascularization, myocardial infarction, admission for heart failure or all‐cause death, was considered the primary outcome.

**Results:**

Of the 4582 patients, 562 (12.3%) had current prescriptions for PDE5 inhibitors before the procedure and 4020 (87.7%) did not. The incidence of MACE was 171 (30.4%) among patients of the PDE5 inhibitor group versus 1482 (36.9%) in the non‐PDE5 inhibitor group (*p* = 0.003). In a propensity score‐matched analysis of 1124 of patients, 171 (30.4%) patients in the PDE5i group and 175 (31.1%) patients in the non‐PDE5i group had a MACE (*p* = 0.84). On multivariable analysis, the treatment with PDE5 inhibitors was not significantly associated with the risk of MACE (odds ratio [OR] = 0.99, 95% CI 0.93–1.06; *p* = 0.86).

**Conclusion:**

In this cohort of veterans undergoing coronary angiography/cardiac catheterization, chronic PDE5i therapy was not associated with an increased risk of MACE at 1 year.

## 1 Introduction

Cardiovascular disease is the global leading cause of death. The World Health Organization (WHO) estimated the number deaths in 2016 from cardiovascular disease to be approximately 18 million people, and lifetime risk of cardiovascular disease exceeds 60% [1]. In the United States, about 700,000 people die yearly of cardiovascular disease, resulting in an estimated annual economic burden of over $200 billion [2, 3].

The coexistence of cardiovascular disease and erectile dysfunction (ED) is common likely due to both disease processes being driven, at least in part, by vascular dysfunction. The resultant high frequency of patients with cardiovascular disease being prescribed phosphodiesterase‐5 (PDE5) inhibitors for ED has driven efforts to identify the effects of PDE5 inhibitors on the cardiovascular system and clinical trials have studied the role of PDE5 inhibitors in cardiac disease [4, 5, 6, 7].

PDE5 inhibition has shown evidence of cardio‐protection in some but not all preclinical models [8, 9, 10]. Chronic administration of PDE5 inhibitors has shown promising results in reducing preclinical adverse cardiac outcomes [11, 12, 13]. However, these findings have not translated consistently as treatment for patients with congestive heart failure (CHF), myocardial infarction, or ventricular arrhythmia [5, 14, 15, 16, 17]. Therefore, despite well‐established safety and tolerance of PDE5 inhibitors, the unclear role of these medications in clinical cardiac pathologies has contributed to the lack of indications for prescribing PDE5 inhibitors in the treatment of cardiovascular disease [18] and has led to an insert package warning about potential risks in patients with coronary disease. Accordingly, we studied the effects of PDE5 inhibitors on the long‐term outcomes of patients referred for cardiac catheterization laboratory procedures.

## 2 Methods

### 2.1 Study Population

This was a retrospective chart review study done at a single Veterans Administration Medical Center, which serves as a referral center for several other medical centers. The study used the Veterans Healthcare Administration electronic medical records (CPRS) and was approved by the institutional medical board for exemption of consent.

We studied a cohort of 4582 consecutive patients who underwent coronary or peripheral angiography or intervention. All patients who underwent the procedures were included. This was a retrospective study and the follow‐up tests were ordered at the discretion of the physician providing care. Of the 4582 patients included, 562 patients (12.3%) had a PDE5 inhibitor prescription before the procedure and 4020 (87.7%) did not.

### 2.2 Outcomes

The primary outcome of this study was the composite incidence of major adverse cardiovascular events (MACE) at 1 year defined as urgent revascularization, myocardial infarction, admission for heart failure, or all‐cause death. The secondary endpoint was all‐cause death as a component of the composite primary outcome.

### 2.3 Statistics

A propensity score was calculated to determine the likelihood that a patient would receive PDE5 inhibitors based on a non‐parsimonious model that included variables such as age, gender, baseline creatinine and other baseline comorbid conditions such as CHF, and diabetes mellitus (DM), as well as medications such as angiotensin converting enzyme inhibitors (ACEI), beta blockers, statins, aspirin or metformin. A propensity score paired analysis was done to reduce the biases that are inherent with retrospective studies. As a sensitivity analysis, the propensity score was used as a covariate in the multivariate analysis. Odds ratios (OR) associated with the primary outcomes were calculated for individual exposures. Kaplan−Meier survival curves were made for the PDE5 inhibitors and no PDE5 inhibitors patient groups at 12 months using the data collected on deaths and other clinical outcomes.

## 3 Results

### 3.1 Study Population

Baseline characteristics of the study population are presented (Table 1). Briefly, this veteran population was predominantly male. The average age was 65 years, 37% of the patients were African American and the mean body mass index (BMI) was 31 kg/m^2^. The average baseline creatinine was 1.4 mg/dL (Table 2). The vast majority of patients had the diagnosis of hypertension (HTN), while approximately half of the cohort had the diagnoses of coronary artery disease (CAD) and/or DM. Approximately 20% of the study population had a history of CHF and/or chronic kidney disease (CKD).

**Table 1:** Baseline characteristics of the (entire) study population.

	Overall	PDE5 inhibitor	No PDE5 inhibitor	*p* value
	*N* = 4582	*N* = 562 (12.3%)	*N* = 4020 (87.7%)	
Male (%)	4416 (96.4)	561 (99.8)	3855 (95.9)	< 0.001
Age (mean [SD])	64.72 (9.71)	62.40 (8.18)	65.04 (9.86)	< 0.001
BMI (mean [SD])	30.83 (6.61)	31.15 (6.10)	30.79 (6.68)	0.22
African American (%)	1694 (37.0)	300 (53.4)	1394 (34.7)	< 0.001
Creatinine (mean [SD] [mg/dL])	1.42 (1.40)	1.47 (1.57)	1.42 (1.37)	0.40
CAD (%)	2463 (53.8)	169 (30.1)	2294 (57.1)	< 0.001
HTN (%)	3783 (82.6)	467 (83.1)	3316 (82.5)	0.76
AFIB (%)	530 (11.6)	51 (9.1)	479 (11.9)	0.05
CHF (%)	984 (21.5)	104 (18.5)	880 (21.9)	0.07
DM (%)	2157 (47.1)	279 (49.6)	1878 (46.7)	0.20
PVD (%)	449 (9.8)	31 (5.5)	418 (10.4)	< 0.001
COPD (%)	871 (19.0)	81 (14.4)	790 (19.7)	0.004
CKD (%)	860 (18.8)	84 (14.9)	776 (19.3)	0.01
*Medications*				
ASA (%)	3554 (77.6)	428 (76.2)	3126 (77.8)	0.42
Plavix (%)	936 (20.4)	61 (10.9)	875 (21.8)	< 0.001
Beta Blocker (%)	2963 (64.7)	299 (53.2)	2664 (66.3)	< 0.001
ACEI/ARB (%)	2503 (54.6)	303 (53.9)	2200 (54.7)	0.75
Statin (%)	3316 (72.4)	405 (72.1)	2911 (72.4)	0.90
Insulin (%)	1004 (21.9)	121 (21.5)	883 (22.0)	0.85
Metformin (%)	952 (20.8)	139 (24.7)	813 (20.2)	0.01
*Procedural details*				
Indication				
Chest pain (%)	3042 (66.4)	372 (66.2)	2670 (66.4)	0.09
CHF/cardiomyopathy (%)	673 (14.7)	86 (15.3)	587 (14.6)	
ACS (%)	538 (11.7)	53 (9.4)	485 (12.1)	
Other (%)	329 (7.2)	51 (9.1)	278 (6.9)	
Findings				
Non‐obstructive CAD (%)	1899 (41.4)	287 (51.1)	1612 (40.1)	< 0.001
Significant CAD (%)	2683 (58.6)	275 (48.9)	2408 (59.9)	
Treatment				
CT Surgery (%)	338 (7.4)	41 (7.3)	297 (7.4)	0.07
PCI (%)	1076 (23.5)	105 (18.9)	971 (24.1)	
Medical (%)	3168 (69.1)	416 (73.8)	2752 (68.5)	

Abbreviations: ACEI/ARB, angiotensin conversion enzyme inhibitor/angiotensin receptor blocker; ACS, acute coronary syndrome; AFIB, atrial fibrillation; ASA, aspirin; BMI, body mass index; CAD, coronary artery disease; CHF, congestive heart failure; CKD, chronic kidney disease; COPD, chronic obstructive pulmonary disease; CT, cardiothoracic; DM, diabetes mellitus; HTN, hypertension; PCI, percutaneous coronary intervention; PDE5, phosphodiesterase 5; PVD, peripheral vascular disease; SD, standard deviation.

**Table 2:** Primary and secondary outcomes in the whole study population.

	Overall	PDE5 inhibitor	No PDE5 inhibitor	*p* value
	*N* = 4582	*N* = 562 (12.3%)	*N* = 4020 (87.7%)	
MACE at 12 months (%)	1653 (36.1)	171 (30.4)	1482 (36.9)	0.003
Death at 12 months (%)	289 (6.3)	19 (3.4)	270 (6.7)	0.003

Abbreviations: MACE, major adverse cardiovascular events; PDE5, phosphodiesterase 5.

Approximately 75% of the study population, regardless of concurrent PDE5 inhibitor use, were on home aspirin and insulin. The majority of patients were on home beta blocker and ACEI/angiotensin receptor blocker (ARB). In addition, approximately 20% of the overall study population were on home clopidogrel and metformin. Chest pain/angina (usually with a positive stress test) was the primary indication for cardiac catheterization in this study, while 12% of patients had signs or symptoms of acute coronary syndrome with or without ST elevation. Nearly 60% of patients were found to have significant CAD, and 24% of patients underwent PCI. The average volume of contrast used was 130 ml. In the propensity score matched cohort, the size of the patient cohort was smaller (1164 patients) but the groups (562 patients each) were well‐matched allowing for a valid comparison of the effects of treatment with PDE5 inhibitors (Table 2).

### 3.2 Effect of PDE5 Inhibitors on MACE and Other Long‐Term Outcomes

The composite incidence of MACE, defined as urgent revascularization, myocardial infarction, hospital admission for heart failure, or all‐cause death, was 30.4% in the PDE5 inhibitor group compared to 36.9% in the non‐PDE5 inhibitor group (171 vs 1482 patients, respectively; *p* = 0.003; Table 3). The secondary outcome measure of death from all causes occurred in 3.4% of the PDE5 inhibitor group compared to 6.7% of the non‐PDE5 inhibitor group (19 vs. 270 patients, respectively; *p* = 0.003; Table 3).

**Table 3:** Baseline characteristics and procedural details of the propensity‐matched study population.

	Overall	PDE5 inhibitor	No PDE5 inhibitor	*p*
	*N* = 1124	*N* = 562	*N* = 562	
Male (%)	1089 (96.9)	561 (99.8)	528 (94.0)	< 0.001
Age (mean [SD])	62.55 (9.10)	62.40 (8.18)	62.69 (9.94)	0.59
BMI	31.38 (6.61)	31.15 (6.10)	31.61 (7.09)	0.25
African American (%)	521 (46.4)	300 (53.4)	221 (39.3)	< 0.001
CAD (%)	339 (30.2)	169 (30.1)	170 (0.2)	> 0.99
HTN (%)	940 (83.6)	467 (83.1)	473 (84.2)	0.68
AFIB (%)	106 (9.4)	51 (9.1)	55 (9.8)	0.75
CHF (%)	211 (18.8)	104 (18.5)	107 (19.0)	0.87
DM (%)	561 (49.9)	279 (49.6)	282 (50.2)	0.90
PVD	60 (5.3)	31 (5.5)	29 (5.2)	0.89
COPD (%)	173 (15.4)	81 (14.4)	92 (16.4)	0.40
CKD (%)	171 (15.2)	84 (14.9)	87 (15.5)	0.86
*Medications*				
ASA (%)	854 (76.0)	428 (76.2)	426 (75.8)	0.94
Plavix (%)	136 (12.1)	61 (10.9)	75 (13.3)	0.23
Beta blocker (%)	586 (52.1)	299 (53.2)	287 (51.1)	0.51
ACEI/ARB (%)	604 (53.7)	303 (53.9)	301 (53.6)	0.95
Statin (%)	815 (72.5)	405 (72.1)	410 (73.0)	0.78
Insulin (%)	255 (22.7)	121 (21.5)	134 (23.8)	0.39
Metformin (%)	280 (24.9)	139 (24.7)	141 (25.1)	0.94
*Procedural details*				
Indication (%)				
Chest pain	761 (67.7)	372 (66.2)	389 (69.2)	0.63
CHF/cardiomyopathy	158 (14.1)	86 (15.3)	72 (12.8)	
ACS	106 (9.4)	53 (9.4)	53 (9.4)	
Other	99 (8.8)	51 (9.1)	48 (8.5)	
Findings (%)				
Non‐obstructive CAD	577 (51.2)	288 (51.1)	289 (51.5)	0.90
Significant CAD	547 (48.7)	275 (48.9)	272 (48.4)	
Treatment (%)				
CT Surgery	88 (7.8)	41 (7.3)	47 (8.4)	0.67
PCI	200 (17.8)	106 (18.9)	94 (16.7)	
Medical	836 (74.4)	413 (73.9)	421 (74.9)	

Abbreviations: ACEI/ARB, angiotensin conversion enzyme inhibitor/angiotensin receptor blocker; ACS, acute coronary syndrome; AFIB, atrial fibrillation; ASA, aspirin; BMI, body mass index; CAD, coronary artery disease; CHF, congestive heart failure; CKD, chronic kidney disease; COPD, chronic obstructive pulmonary disease; CT, cardiothoracic; DM, diabetes mellitus; HTN, hypertension; PCI, percutaneous coronary intervention; PDE5, phosphodiesterase 5; PVD, peripheral vascular disease; SD: standard deviation.

**Figure 1:**
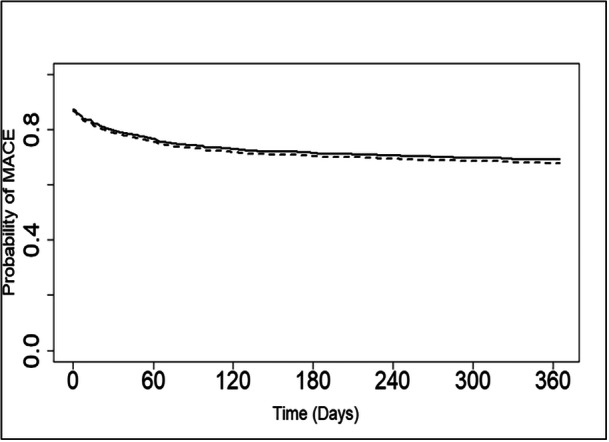
Kaplan−Meier curves showing no difference in major adverse cardiac events (*p* = 0.85) of patients following cardiac catheterization who were on chronic PDE5 inhibitor therapy (solid line) compared to patients who were not on chronic PDE5 inhibitor therapy (dashed line).

In the multivariable analysis (Table 4, Figure 1), there was no significant association between PDE5 inhibitor therapy and the risk of MACE at 12 months. However, there was a significant association between PDE5 inhibitor treatment and a decreased risk of death at 12 months (HR 0.56; 95% CI 0.31, 0.99; *p* = 0.04). The hazard ratios of the various baseline characteristics and 12‐month incidence of MACE revealed that age and a history of DM were significantly associated with an increased incidence of MACE (Table 4), while a h. We tested for the interaction between PDE5i intake and CHF, between PDE5i intake and CAD, and between PDE5i intake and CKD, with the outcome being MACE, and the interaction was not statistically significant in any of the tests. We also tested the same interactions between PDE5i intake and CHF, CAD, and CKD, with the outcome being survival at 12 months, and the interactions were not statistically significant in any of these tests either. Central Illustration 1.

**Table 4:** Outcomes on multivariable analysis in the propensity matched population.

Outcome	Predictor	Odds ratio	95% CI	*p* value
MACE at 12 months				
	PDE5i	0.99	0.93, 1.06	0.86
	Age	1.01	1.00, 1.01	< 0.001
	CHF	0.90	0.83, 0.98	0.01
	CKD	1.03	0.93, 1.13	0.60
	DM	1.09	1.02, 1.17	0.01
Death at 12 months				
	PDE5i	0.56	0.31, 0.99	0.04
	Age	1.07	1.03, 1.1	< 0.001
	CHF	2.53	1.38, 4.65	0.002
	CKD	1.34	1.18, 4.34	0.01
	DM	1.34	0.7, 2.55	0.37
	Beta blocker	0.81	0.46, 1.45	0.48
	Statin	0.63	0.34, 1.16	0.13

Abbreviations: ACEI/ARB, angiotensin conversion enzyme inhibitor/angiotensin receptor blocker; AFIB, atrial fibrillation; ASA, aspirin; CHF, congestive heart failure; CKD, chronic kidney disease; DM, diabetes mellitus; HTN, hypertension; MACE, major adverse cardiovascular events; PDE5i, phosphodiesterase 5 inhibitor.

**Central Illustration 1:**
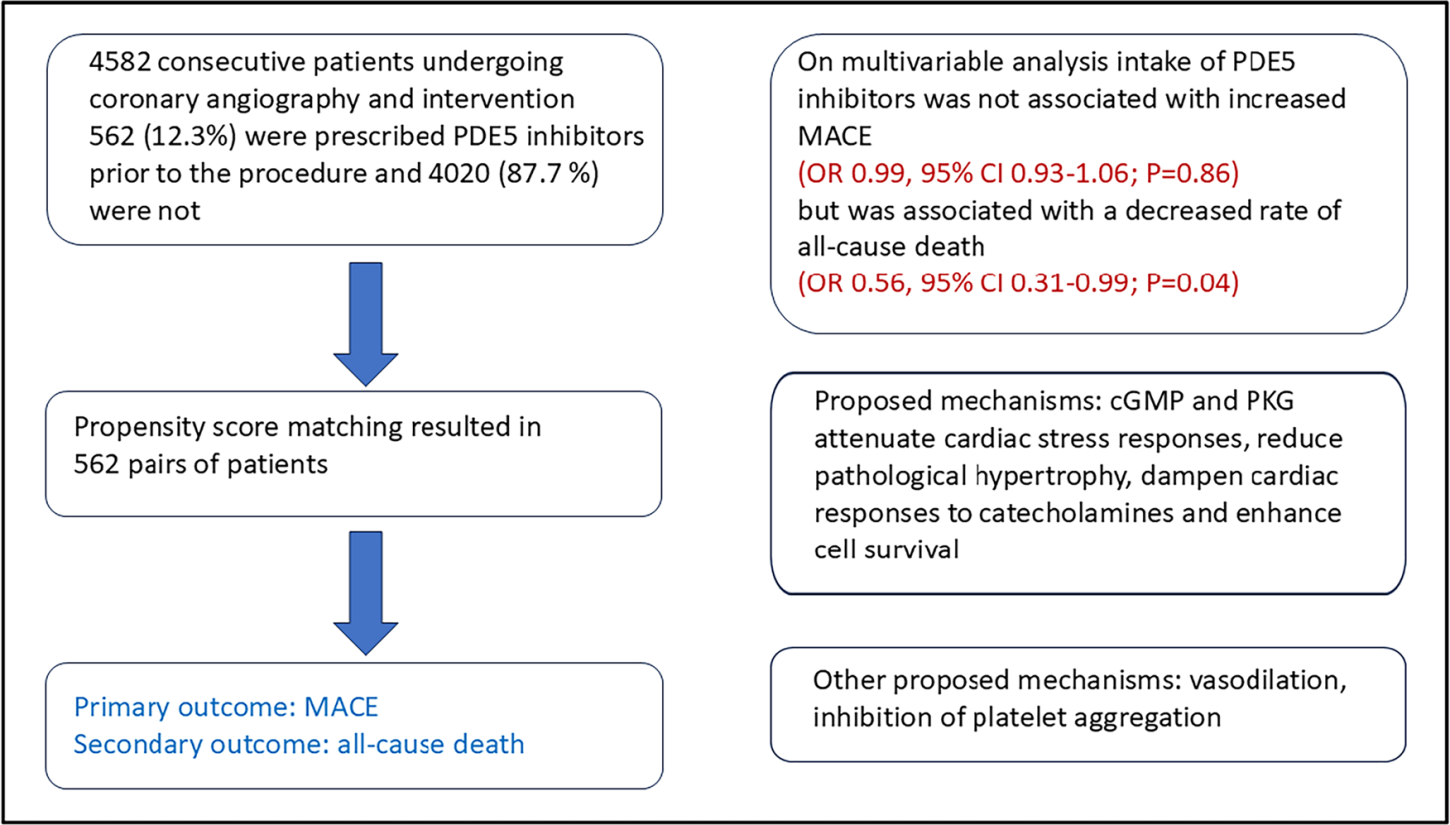
cGMP, cyclic guanidyl monophosphate; MACE, major adverse cardiovascular events; PDE5, phosphodiesterase 5; PKG, protein kinase G. [Color figure can be viewed at wileyonlinelibrary.com]

## 4 Discussion

The frequent concurrence of cardiovascular disease and ED has led to a significant number of patients on PDE5 inhibitors who ultimately require coronary angiography and cardiac catheterization. This study was designed to investigate the association between PDE5 chronic therapy before coronary angiography/cardiac catheterization and MACE. Our data suggests that veterans on PDE5 inhibitors who undergo cardiac catheterization do not have an increased risk of MACE at 1 year. At the same time the data shows that there is an association between a reduced risk of all cause death and the intake of PDE5 inhibitors (Table 5).

**Table 5:** Salient points.

Patients with cardiovascular disease are often denied therapy with PDE5 inhibitors because of concerns for cardiovascular risks We studied the outcomes of patients with cardiovascular disease who underwent coronary angiography and interventions and who had prescriptions for PDE5 inhibitors. We found no increase in major adverse cardiovascular events in patients who had prescriptions for PDE5 inhibitors We found that the patients who had PDE5 inhibitors prescriptions had a reduced all‐cause mortality

The overall effect of PDE5 inhibition in myocardial ischemia seems to be beneficial, but evidence is largely preclinical with inconsistent results in the clinical setting. Early studies in animal models showed improved ventricular recovery, decreased myocardial infarction, and reduced ventricular infarct size following ischemia‐reperfusion injury and PDE5 inhibition [9, 19]. Findings from a meta‐analysis suggested that chronic PDE5 inhibitor use imparts a beneficial cardiac inotropic effect together with anti‐remodeling properties across different populations, suggesting that patients with longstanding cardiac disease could benefit from PDE5 inhibition‐induced cardio‐protection [4]. However, in another study, the effect of PDE5 inhibition on exercise tolerance times was neutral in patients with stable CAD [16]. Taken together, there is some evidence that PDE5 inhibition could be beneficial in ischemic coronary disease [7]. Our study is a retrospective study that did not investigate the mechanisms that could lead to improved outcomes in cardiac patients who are prescribed PDE5 inhibitors. However, proposed mechanisms are the activation of cGMP and PKG which can attenuate cardiac stress responses, reduce pathological hypertrophy, dampen cardiac responses to catecholamines and enhance cell survival. Other proposed mechanisms are vasodilation and inhibition of platelet aggregation [7, 20, 21],

A study in Swedish men with first myocardial infarction found that treatment with PDE5 inhibitors was associated with a lower risk of death and cardiovascular events [22]. This study had a control group which was not receiving any treatment for ED, potentially resulting in confounding for indication. This led to a recent subsequent study investigating the association between PDE5 inhibition versus prostaglandin E1 in men with stable CAD [23]. Results from this study showed that in men with stable CAD, treatment with PDE5 inhibitor was associated with lower cardiovascular outcomes including death, myocardial infarction, heart failure, and revascularization [23]. Because the study was observational, no inferences on causality can be made.

Findings from the present study offer an additional perspective of potential benefit from chronic PDE5 inhibition. Together with current literature, our findings support the notion that PDE5 inhibition is not deleterious and could be beneficial in treating patients with cardiovascular disease. Though the mechanism remains unclear as to how PDE5 inhibition could result in a reduction in all‐cause death, these findings are significant because of the widespread use of PDE5 inhibitors for the treatment of ED, as well as common use in advanced pulmonary hypertension [24], and off‐label use in the management of high‐altitude pulmonary edema [25], Raynaud phenomenon [26], and colorectal cancer as adjuvant therapy [27]. Interestingly, a recently published meta‐analysis suggests that intake of PDE5 inhibitors by patients without known cardiovascular disease is associated with reduced risks of both MACE and all cause death [28]. However, our study population was different because it had known or suspected cardiovascular disease of sufficient severity to warrant a cardiac catheterization/coronary angiography. This may account for the better results of the meta‐analysis compared to the results of our study. Finally, a recently published study in a large US based hospital database found a reduction in all‐cause death (as well as stroke and venous thromboembolism) in patients taking PDE5 inhibitors, consistent with our findings [29].

Limitations of this study include the retrospective design of the study. Because of this, the findings can only be considered as hypothesis‐generating. In addition, because this study was conducted at a Veterans Administration Medical Center, the population investigated was predominantly male and may be slightly older than the typical population of patients undergoing cardiac catheterization. Because of this, extrapolation to female and younger patients should be done with caution. Another limitation is the fact that we do not have information about how many patients were actually taking the PDE5i since the data that we collected pertains to prescriptions only. However, if only a small fraction of patients were indeed taking the medications prescribed this would skew the results more toward the null hypothesis. There is also incertitude regarding how many tablets the patients were taking and whether there is a chronic effect of PDE5i in patients who take the medication on an as needed basis. The VA offers four doses per month free of charge to the veterans as part of the pharmacy benefits, but we do not have information about how the tablets were taken. However, a study investigating the intake of PDE5 inhibitors in a random sample of 24,745 veterans over 66 years of age [30] showed that most of them who filled a prescription for PDE5i only filled it through VA pharmacies and only 3% were also getting prescriptions through Medicare.

## 5 Conclusion

In conclusion, our study showed that in a population of United States veterans undergoing coronary angiography/cardiac catheterization, chronic intake of PDE5 inhibitors was not associated with a significantly reduced rate of MACE but was associated with a significantly reduced risk of death at 1 year after the procedure. Further prospective, large‐scale trials are needed to confirm whether starting therapy with PDE5 inhibitors in patients undergoing coronary angiography/cardiac catheterization can improve long‐term outcomes in these patients.

## Conflicts of Interest

The authors declare no conflicts of interest.

## Data Availability

Data are obtained from the electronic medical records system (CPRS) that is used throughout the integrated healthcare system. These data are not publicly available and are limited to groups operating on behalf of the Department of Veterans Affairs.
